# Manipulating Cardiomyocyte Plasticity for Heart Regeneration

**DOI:** 10.3389/fcell.2022.929256

**Published:** 2022-07-11

**Authors:** Toshiyuki Ko, Seitaro Nomura

**Affiliations:** Department of Cardiovascular Medicine, Graduate School of Medicine, The University of Tokyo, Tokyo, Japan

**Keywords:** heart failure, myocardial infarction, reprogramming, regeneration, cardiomyocyte proliferation

## Abstract

Pathological heart injuries such as myocardial infarction induce adverse ventricular remodeling and progression to heart failure owing to widespread cardiomyocyte death. The adult mammalian heart is terminally differentiated unlike those of lower vertebrates. Therefore, the proliferative capacity of adult cardiomyocytes is limited and insufficient to restore an injured heart. Although current therapeutic approaches can delay progressive remodeling and heart failure, difficulties with the direct replenishment of lost cardiomyocytes results in a poor long-term prognosis for patients with heart failure. However, it has been revealed that cardiac function can be improved by regulating the cell cycle or changing the cell state of cardiomyocytes by delivering specific genes or small molecules. Therefore, manipulation of cardiomyocyte plasticity can be an effective treatment for heart disease. This review summarizes the recent studies that control heart regeneration by manipulating cardiomyocyte plasticity with various approaches including differentiating pluripotent stem cells into cardiomyocytes, reprogramming cardiac fibroblasts into cardiomyocytes, and reactivating the proliferation of cardiomyocytes.

## Introduction

**FIGURE 1:**
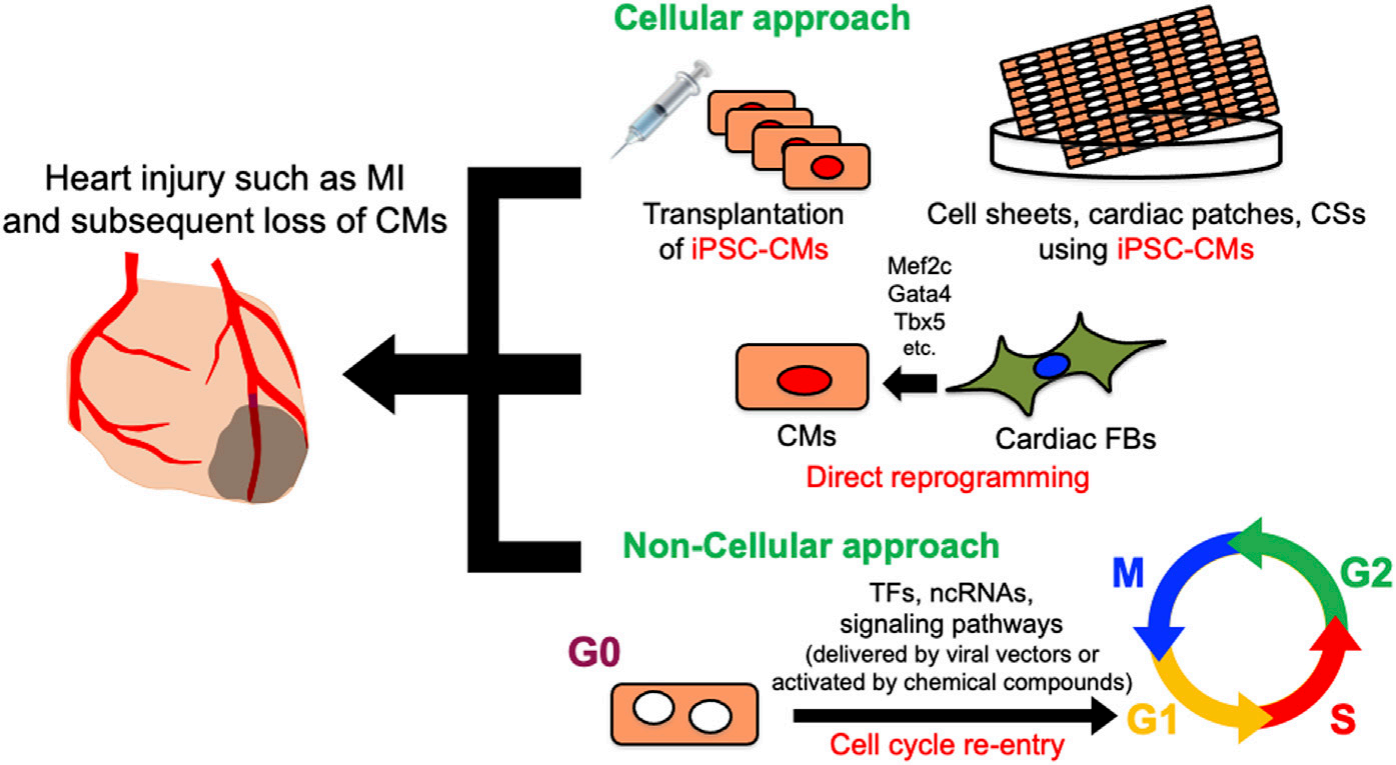
Schematic diagram of cellular and non-cellular approach for heart regeneration therapy. Cellular approach includes iPSC-CMs and CMs derived from cardiac FBs by direct reprogramming. In non-cellular approach, various genes, ncRNAs, and signaling pathways promote heart regeneration by inducing cardiomyocyte cell cycle re-entry. CMs, Cardiomyocytes; CFs, cardiac fibroblasts; CSs, cardiac spheroids; iPSC-CMs, induced pluripotent stem cell-derived cardiomyocytes; TFs, transcription factors; ncRNAs, non-coding RNAs.

Despite decades of development of therapeutic approaches, cardiovascular disease remains the leading cause of morbidity and mortality worldwide, accounting for an estimated 18.6 million deaths annually in the United States (Aparicio et al., 2021). Along with the brain, the adult mammalian heart is arguably one of the least regenerative organs, and cardiomyocytes (CMs) are considered to be terminally differentiated. CMs account for approximately 75% of the left ventricular volume in healthy adults (Vliegen et al., 1991). They facilitate the pumping of blood into the circulatory system by coordinating contraction and diastole. Therefore, any massive injury to the heart will induce progression to heart failure, resulting in the death of CMs, which become replaced with fibrotic scar tissue (Fu et al., 2018; Le Bras, 2018; Yokota et al., 2020). One of the most common pathological heart injuries is myocardial infarction, which is accompanied by the massive irreversible loss of CMs. Since mature CMs have little regenerative capacity, the remaining CMs cannot fully proliferate and restore lost cells. Compensatory scarring to replace dead tissue with cardiac fibroblasts leads to heart remodeling, which ultimately reduces cardiac systolic function and induces heart failure and sudden cardiac death (Jenča et al., 2021). Therapies for heart failure have decreased mortality, but evidence has yet to support the notion that CMs can regenerate or that lost CMs can be replaced in patients who undergo such therapies. Although heart transplantation is feasible and effective for advanced heart failure (Chambers et al., 2021), donor hearts are scarce and multiple post-transplant complications have limited their application. Therefore, heart regeneration has attracted interest as a novel approach to treating heart failure. The approaches to heart regeneration currently comprise differentiating pluripotent stem cells (PSCs) into CMs, reprogramming cardiac fibroblasts (CFs) into CMs, and reactivating the proliferation of surviving CMs (Figure 1). This review introduces recent innovations in heart regeneration and discusses its prospects.

### Heart Regeneration Using Pluripotent Stem Cells

Human PSCs consist mainly of embryonic (ESCs) and induced pluripotent (iPSCs) stem cells. Cellular reprogramming and iPSC generation were discovered in 2007 (Takahashi et al., 2007). Several approaches have since been used to differentiate iPSCs into CMs (iPSC-CMs). Transplanted iPSC-CMs improved cardiac function and survival after myocardial infarction in animal models (Shiba et al., 2016; Park et al., 2019). In addition, recent studies have already established tumorigenicity assay system to detect malignantly transformed cells within iPSC-CMs (Sougawa et al., 2018; Ito et al., 2019). Transplanted iPSC-CMs can trigger an immune response mediated by natural killer cells, resulting in low engraftment (Nakamura et al., 2019), and immature iPSC-CMs might lead to ventricular tachycardia (Shiba et al., 2016). Differentiation methods described so far have been limited by the inability to generate mature CMs, which are differ from fetal CMs in terms of function, morphology, and electrophysiology (Gomez-Garcia et al., 2021).

Several studies have attempted to improve the low engraftment rate of transplanted cells and enhance the maturity of iPSC-CMs (Protze et al., 2019). Tissue engineering has improved the engraftment rate and therapeutic effects of transplanted cells using scaffolds such as hydrogels (Chow et al., 2017), cell sheets (Kawamura et al., 2017), cardiac patches (Gao et al., 2018; Zhu D. et al., 2021; Querdel et al., 2021), and three-dimensional bio-printed tissues (Gao et al., 2017; Kawai et al., 2022). Activating Wnt/β-catenin signaling promotes human PSC differentiation and the production of high-purity (up to 98%) iPSC-CMs (Lian et al., 2012). A Gsk3β inhibitor combined with Wnt-C59 contributes to the production of high-purity iPSC-CMs (Burridge et al., 2014). Glucose and glutamine depletion eliminates residual undifferentiated human PSCs after CM differentiation (Tohyama et al., 2016). Other recent endeavors to improve the maturity of iPSC-CM include long-term culture (Ebert et al., 2019; Funakoshi et al., 2021), metabolic substrates (Hu et al., 2018; Gentillon et al., 2019; Miklas et al., 2019; Garbern et al., 2020) and miRNAs (Kuppusamy et al., 2015).

In terms of clinical application of iPS-CMs, a clinical trial of allogeneic iPSC-CM sheets for patients with ischemic cardiomyopathy has started (ClinicalTrials.gov, #jRCT2053190081). The safety and efficiency of transplantation of human iPSC-derived cardiac spheroids (CSs) has been shown using swine heart failure model (Kawaguchi et al., 2021). These seem to be a feasible way to improve cardiac function in patients with heart failure. Although PSC-based heart regeneration has potential for treating severe heart failure, considering their immaturity of iPSC-CM and poor survival of transplanted cells, further investigation of its safety and efficacy is still required. From therapeutic perspective, since the differentiation and preparation of iPSC-CMs is a long process, its transplantation will be limited only to the patients with chronic heart failure. Besides, the current intramyocardial transplantation of iPSC-CMs needs open-chest surgery, which will be high risk for the patients with severe heart failure. Development of a less invasive transplant methods such as catheter delivery may be helpful.

### Reprogramming non-CMs for Heart Regeneration

**FIGURE 2:**
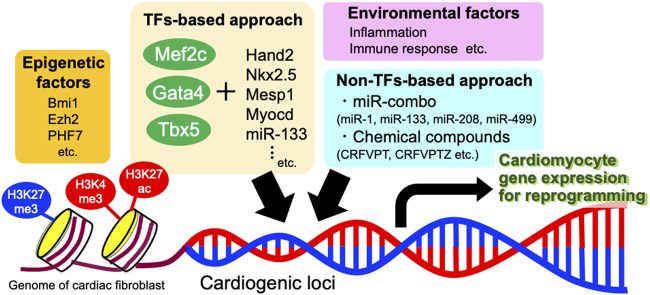
Molecular mechanisms involved in direct reprogramming of cardiac fibroblasts into cardiomyocytes. Major methods include TFs-based approach and non-TFs-based approach. Besides, various epigenetic factors and environmental factors also affect the efficiency of direct reprogramming. TFs, transcription factors; miR, microRNA.

Although CMs account for approximately 75% of the normal myocardial tissue volume, they constitute only 40% of the total cell number (Vliegen et al., 1991). Cardiac fibroblasts are among the most important non-CM components of the heart. Upon myocardial infarction, they are activated and recruited to the injured site to form scar tissue (Tallquist and Molkentin, 2017; Fu et al., 2018). Given that the number of CFs can be increased by the proliferation of resident CFs in injured heart tissue, reprogramming this abundant cell population into functional CMs would be an ideal strategy for heart repair in response to ischemic injury. The process of converting somatic cells from one lineage to another without transitioning through a stem cell state is called direct reprogramming (or transdifferentiation; Figure 2) (Wang H. et al., 2021). Compared with the generation of iPSC-CMs, direct reprogramming of CFs into CMs enables more rapid and efficient conversion of cells *in situ* without the need for cell expansion *ex vivo* and transplantation. Three years after the epoch-making discovery of iPSCs (Takahashi et al., 2007), the combined transcription factors Gata4, Mef2c, and Tbx5 (GMT) were found to directly convert CFs into CM-like cells which show the organized sarcomeric structures, global CM-like gene expression profiles, action potentials, and spontaneous contractions (Ieda et al., 2010). Reprogramming CFs into CMs requires inhibiting fibroblast signatures, extensive chromatin remodeling to overcome existing epigenetic barriers, and the simultaneous acquisition of a CM-like chromatin profile. Several groups then found that adding or modifying core reprogramming factors in addition to GMT such as other transcription factors, kinases, and microRNAs (miRs), might further promote direct reprogramming and CM maturation. Other transcription factors and kinases which enhance the efficiency of reprogramming includes Heart- and Neural Crest Derivatives-Expressed Protein 2 (Hand2) (Song et al., 2012; Nam et al., 2014), NK2 homeobox 5 (Nkx2-5) (Addis et al., 2013), Mesoderm Posterior bHLH Transcription Factor 1 (MESP1) and Myocardin (Wada et al., 2013), Estrogen Related Receptor Gamma (ESRRG), MESP1, MYOCD, and Zinc Finger Protein Multitype (ZFPM) (Fu et al., 2013), and AKT and HAND2 (Zhou et al., 2015). The transcription factor Guanine-Adenine-Thymine-Adenine (GATA) 4 is thought to have the potential for human and mouse cardiac reprogramming, whereas Myocyte-Specific Enhancer Factor 2C (MEF2C) and T-Box Transcription Factor 5 (TBX5) are important for activating cardiac gene expression to initiate CM maturation (Liu et al., 2017; Hashimoto et al., 2019; Stone et al., 2019). Addition of miR-133 to either GMT or GMT + Myocd + Mesp1 promoted CM reprogramming efficiency and maturation (Muraoka et al., 2014). Addition of miR-1 and miR-133 to GMT + HAND2 (GHMT) (Nam et al., 2013) or GMT + MYOCD + NKX2.5 (Christoforou et al., 2017) accelerated CM reprogramming and upregulates cardiac gene expression. Mechanistically, miR-133 directly targets Snai1, a master regulator of epithelial-to-mesenchymal transition, and represses its protein production (Muraoka et al., 2014). The combination of muscle-specific miR-1, miR-133, miR-208, and miR-499 (miR combo) could be an alternative to overexpressing transcription factors for directly reprogramming CFs into CMs (Jayawardena et al., 2012, 2015). The miR combo induces the expression of endogenous reprogramming factors GHMT (Dal-Pra et al., 2017).

As direct reprogramming requires chromatin remodeling to transform CFs into CMs, transcription factors such as GMT must be able to engage genes that are developmentally silenced for expression in fibroblasts. Therefore, epigenetic factors play important roles in promoting or inhibiting cardiac reprogramming. Analyses of epigenetic status have revealed that the inactive heterochromatin-associated histone mark H3K27me3 increases at fibroblast promoters and decreases at cardiac promoters, whereas activated chromatin marks (H3K4me3 and H3K27ac) were enriched at cardiac promoters during direct reprogramming (Fu et al., 2013; Liu et al., 2016; Dal-Pra et al., 2017; Riching et al., 2021). Promoters of the cardiac genes, such as NPPA and MYH6, are similarly demethylated immediately after GMT induction during reprogramming to CMs (Roost et al., 2017). Efforts to improve direct reprogramming have included manipulating epigenetic factors by knockdown of Bmi1 (Zhou et al., 2016) and Ezh2 (Hirai and Kikyo, 2014; Tang et al., 2021) and overexpression of PHF7 (Garry et al., 2021).

Small molecules that enhance the efficiency of direct reprogramming have been added to GMT or GHMT (Zhao et al., 2015; Mohamed et al., 2017). In contrast, fibroblasts have been directly reprogrammed to differentiate into CMs in mice using a cocktail containing CHIR99021, RepSox, Forskolin, Valproic Acid, Parnate, and (E)-4-[2-(5,7,8-tetrahydro-5,5,8,8-tetramethyl-2-naphthyl)-propen-1-yl]benzoic acid (TTNPB) (CRFVPT), and the small molecule combination CRFVPT + DZNep (CRFVPTZ) induced iPSCs from embryonic fibroblasts (Hou et al., 2013; Fu et al., 2015). The Bmi1 inhibitor PTC-209 promotes the efficiency of direct reprogramming using CRFVPT (Testa et al., 2020). A similar protocol showed that a combination of nine compounds that partially overlapped with CRFVPT is sufficient and necessary to induce the direct reprogramming of human fibroblasts (Cao et al., 2016). Upon transplantation into the infarcted hearts of immunodeficient mice, these human fibroblasts were efficiently converted into CMs that expressed CM markers and developed organized sarcomeric structures. However, such chemically converted CMs lack an organized sarcoplasmic reticulum and transverse tubule structures, which are typical of adult CMs, suggesting the electric and mechanical immaturity. Therefore, further investigations are required to establish the optimal chemical conditions for CM maturation.

The local environment might also influence the efficiency of direct reprogramming (Van Handel et al., 2022). High-throughput screening showed that diclofenac enhances cardiac reprogramming by inhibiting cyclooxygenase-2-mediated prostaglandin E2/prostaglandin E receptor 4 signaling; this silences inflammation in aged fibroblasts that express more cyclooxygenase-2 than embryonic fibroblasts (Muraoka et al., 2019). Similarly, unbiased screening of transcription factors uncovered Znf281 as an inducer of direct reprogramming in murine CFs and showed that Znf281 enhanced CM generation by suppressing inflammatory gene expression while modulating cardiac gene expression (Zhou et al., 2017). Collectively, inflammation appears to be an important hurdle for direct reprogramming in mouse. However, there is apparent discrepancy between mouse and human reprogramming because some studies showed that inflammation and immune responses are required for direct reprogramming of human fibroblasts into CMs (Stone et al., 2019; Zhou et al., 2019). In addition to inflammation, previous studies showed that the efficiency of direct reprogramming as well as CM maturation is likely to be more efficient *in vivo* rather than *in vitro*; indicating that the cardiac microenvironment could be a favorable factor for reprogramming (Qian et al., 2012; Song et al., 2012; Jayawardena et al., 2015). Cardiac injury and subsequent myofibroblast activation are essential for CM generation (Qian et al., 2012; Song et al., 2012). Successfully reprogrammed cells exit the cell cycle early, indicating that proliferation might be detrimental to cardiac reprogramming (Liu et al., 2016; Zhou et al., 2019). The stiffness of extracellular environment would also affect the efficiency of direct reprogramming. The polystyrene culture dishes which are used for *in vitro* culture have a hardness of 1 GPa (= 1 × 10^6^ kPa), which is much harder than the heart tissue (around 10 kPa) *in vivo*. Recent report showed that soft extracellular matrix promotes cardiac reprogramming and improves the maturity of generated CMs through the inhibition of YAP/TAZ and suppression of fibroblast signatures (Kurotsu et al., 2020).

Cardiac repair and regeneration *in vivo* are the goals of direct programming using the retroviral or lentiviral delivery of reprogramming cocktails (GMT, GHMT, and miR combo). These cocktails improve cardiac function and reduce fibrosis in a murine myocardial infarction model (Inagawa et al., 2012; Qian et al., 2012; Song et al., 2012; Jayawardena et al., 2015). However, integrative viral vectors, retroviruses, and lentiviruses present major concerns regarding possible mutagenetic effects such as the random genomic integration of virally overexpressed reprogramming factors. Therefore, non-integrative Sendai virus (SeV) vectors have been developed (Ieda et al., 2010). Direct reprogramming using SeV vectors considerably improved cardiac function and reduced fibrosis compared with retroviral vectors (Miyamoto et al., 2018). Direct reprogramming generates CMs mainly through bona fide cardiac reprogramming and not through fusion events between CMs and CFs (Isomi et al., 2021). Recently, nanotechnology-based approaches for direct reprogramming have recently attracted interest (Liu et al., 2021). Several studies have used nanoparticles or fused polyarginine-lipofectamine complexes to generate iPSCs *in vitro* or involved them in direct reprogramming (Zhu et al., 2014; Lee et al., 2015). Cationic gold nanoparticles that can load GMT expression plasmids for direct CF reprogramming recovered cardiac function and reduced fibrosis in a murine model of myocardial infarction (Chang et al., 2019). A similar approach using a nanocarrier to deliver a miR combination resulted in efficient direct reprogramming and recovered cardiac function after myocardial infarction (Yang et al., 2021). Nanoparticles with the natural inflammation-homing ability and high affinity for CFs can specifically target cardiac fibroblasts in the injured heart (Wang H. et al., 2021). Nanoparticles intravenously injected into an infarcted heart delivered the miR combo into fibroblasts and improved cardiac function *via* efficient reprogramming.

Reprogramming CFs into CMs *in vivo* is a powerful and attractive alternative strategy for myocardial regeneration. Compared with transplantation of iPSC-CMs, *in vivo* direct reprogramming is simple and fast, and can be used as a treatment for patients with acute heart failure after myocardial infarction. To date, the considerable effort by numerous research groups has targeted the dissection of direct reprogramming to find effective protocols using transcription factors, miRs, and chemical compounds (Wang Q. et al., 2021). Regardless of substantial progress, further studies are warranted to assess the efficacy and safety of these approaches to heart regeneration before attempting clinical trials. The *in vivo* study using large animals whose heart rate is similar to human will be especially important because incidence of arrhythmias after treatment may be different depending on heart rate. Besides, many *in vivo* direct reprogramming studies showed that generation of CMs from CFs tend to be seen in the ischemic border zone (Qian et al., 2012; Song et al., 2012; Jayawardena et al., 2015). Since CFs are rich in heterogeneity, probability of reprogramming might be different among each population of CFs. Therefore, comprehensive understanding of the cellular characteristics of CFs by single-cell analysis will be important. Furthermore, since all studies of direct reprogramming *in vivo* involved mouse models of myocardial infarction, it is of significant interest whether reprogramming *in vivo* could translate effectively to non-ischemic causes of heart failure.

### Proliferation of Extant CM for Heart Regeneration

**FIGURE 3:**
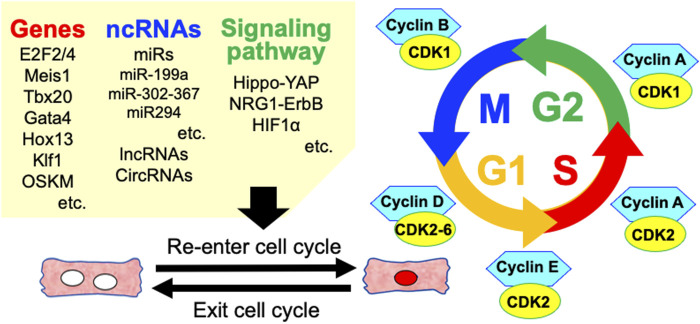
Various factors involved in the regulation of cardiomyocyte cell cycle re-entry. ncRNAs, non-coding RNAs; miRs, microRNAs; lncRNAs, long non-coding RNAs; circRNAs, circle RNAs.

The ability to regenerate damaged myocardial tissue varies amongst vertebrates (Vivien et al., 2016; Velayutham et al., 2019). Some lower vertebrates such as zebrafish and amphibians can regenerate their hearts, but no adult mammalian species can robustly regenerate their hearts. The human heart can produce new adult CMs throughout the life, whereas the rate of CM division is very low (Lázár et al., 2017). However, fetal and early neonatal mammals have such abilities (Lam and Sadek, 2018). In addition, terminally differentiated myocytes can re-enter the cell cycle and divide through manipulation of the CM cell cycle, signaling pathways, miRs, and environmental factors. Inducing CM cell-cycle entry and heart repair is emerging as an effective strategy to compensate for lost functional CMs and improve impaired heart function (Figure 3).

### Intervention Using Cell Cycle Genes

Cyclins and their related kinases (CDKs) regulate the cell-cycle transition phase from G1 to S, induce DNA synthesis, and maintain cell-cycle activity. More specifically, cyclins A/D/E are involved in DNA synthesis, whereas cyclin B mainly regulates cytokinesis. In contrast, complexes of cyclins/CDKs in adult mammalian CMs are constitutively inhibited by the CIP/KIP family (p21, p27, and p57) and the INK4 family (p16, p15, p18, and p19). Since the decline in the proliferative capacity of post-mitotic CMs might be due to the downregulation of cyclins/CDKs and upregulation of CIP/KIP family and INK4 family, whether the intervention with these cell-cycle regulators might induce CM cell-cycle entry has been widely investigated (Salama et al., 2021). For example, overexpression of cyclin A2 in murine and porcine infarcted hearts improved heart function, and activated the cell cycle in CMs (Woo et al., 2006; Cheng et al., 2007; Shapiro et al., 2014). Inhibition of the CIP/KIP family increased expression of cyclins and reactivated the cell cycle in CMs (Di Stefano et al., 2011). Inactivating Retinoblastoma 1 (RB1) and cyclin-dependent kinase inhibitor 2a (CDKN2A) or downregulating p21 can induce CMs to enter mitosis and synthesize DNA (Hatzistergos et al., 2019; Volland et al., 2020). The roles of other cyclins and CDKs (such as cyclin D1, D2, B1, CDK2, and CKD4) in the re-entry of CMs into the cell cycle have been investigated. However, reactivating CM cell cycle activity and promoting DNA synthesis alone results in multinucleation or polyploidy without complete CM cytokinesis. Combinations of various cell-cycle genes may be more efficient in activating the cell cycle in CMs. Overexpressing the cell-cycle genes CDK1, CDK4, cyclin B1, and cyclin D1 (4F) induced stable cell cycle division (determined by EdU and PHH3) in 15%–20% of the adult mouse CMs, rat CMs, and iPSC-CMs, which robustly completed cytokinesis *in vivo* (Mohamed et al., 2018). A polycistronic non-integrating lentivirus encoding 4F, in which each of the four factors was driven by CM-specific Troponin T2 significantly improved systolic function and reduced the size of scars after ischemic reperfusion in rats and pigs (Abouleisa et al., 2022). These findings indicated that modulating cell-cycle components is a promising strategy for unlocking the proliferative potential of CMs in the injured adult heart.

### Intervention With Transcription Factors

The cell cycle is controlled by a complex regulatory network, and several cell-cycle-associated transcription factors such as the E2F family, T-Box Transcription Factor 20 (Tbx20), and Myeloid ecotropic viral integration site 1 (Meis1) have been verified as potential initiators or drivers that could promote mammalian CM cell-cycle re-entry.

The E2F family includes a series of transcription factors that can bind to the adenovirus E2 promoter and activate viral genes. These factors play fundamental roles in DNA replication and cell cycle progression by regulating the expression of several cell cycle-associated genes. Overexpression of E2F2 in mice significantly increased the number of Bromodeoxyuridine (BrdU), Phosphohistone H3 (PHH3), and Aurora kinase-positive CM nuclei, suggesting that E2F2 not only induces DNA synthesis, but also promotes CMs to complete the entire cell cycle (Ebelt et al., 2008). The regulatory pocket protein of E2F, Rb combines with E2F2 and inhibits its activation. Phosphorylation of Rb by CDK2 and CDK4 induces Rb dissociation from E2F2, thus triggering the expression of DNA synthesis-associated genes and leading to increased CM cycling (MacLellan et al., 2005; Sdek et al., 2011). Furthermore, E2F2 suppresses the expression of pro-apoptotic genes in CMs (Ebelt et al., 2005) and E2F4 is required during the G2/M phase through the induction of cyclins A and E (Van Amerongen et al., 2010).

T-box family transcription factor 20, is essential for early heart development and adult heart function in various organisms. Deletion of Tbx20 resulted in embryogenic death during mid-gestation (Chakraborty et al., 2013). A Tbx20 mutation causes CM arrest at the G1/S phase transition and reduces proliferation in zebrafish. In contrast, overexpression of Tbx20 enhanced the proliferation of differentiated CMs, resulting in an enlarged heart with significantly more CMs (Lu et al., 2017). Consistently inducible CM-specific Tbx20 KO in adult mice resulted in the onset of severe cardiomyopathy accompanied by arrhythmias and death within 1–2 weeks of Tbx20 ablation (Shen et al., 2011). In contrast, overexpression of Tbx20 promoted mature CM proliferation in mice and resulted in increased numbers of small, mononucleated CMs by activating the BMP2/pSmad1/5/8 and PI3K/AKT/GSK3β/β-catenin signaling pathways (Chakraborty et al., 2013), while repressing expression of the cell-cycle inhibitors p21, Meis1, and B-cell translocation gene 2 (Btg2) (Xiang et al., 2016). T-Box Transcription Factor 20 participates in the regulation of angiogenesis through the signaling cascade of Tbx20-PROK2-PROKR1 (Meng et al., 2018). Like Tbx20, Tbx6 also promotes CM proliferation by upregulating multiple cell-cycle activators and suppressing Rb1 (Haginiwa et al., 2019).

The essential regulator of heart development Meis1 belongs to the three-amino acid loop extension (TALE) homeobox gene family (Paige et al., 2012; Wamstad et al., 2012) and it limits the postnatal proliferation capacity of CMs. Abundant Meis1 expression in P7 CMs correlates with postnatal cell-cycle arrest (Mahmoud et al., 2013). The CM proliferative capacity in postnatal mice with Meis1 KO was extended for up to 14 days without deleterious effects on cardiac function or induced hypertrophy, whereas overexpression of Meis1 inhibited neonatal cell proliferation and neonatal heart regeneration. Mechanistically, Meis1 regulates transcription of the CDK inhibitors p15, p16, and p21 (Mahmoud et al., 2013) and regulates cardiac metabolism. Knockdown of Meis1 in cultured CMs increased respiratory capacity and mitochondrial activity while decreasing glycolytic gene expression (Lindgren et al., 2019).

Other transcription factors that promote CM cell-cycle entry include Gata4 (Malek Mohammadi et al., 2017) and Hox13 (Nguyen et al., 2020). Some studies have focused on inducing CM dedifferentiation to promote cell proliferation. The induction of Klf1 directed epigenetic reprogramming of the cardiac transcription factor network in the injured adult zebrafish myocardium and permited coordinated CM dedifferentiation and proliferation (Ogawa et al., 2021). The CM-specific expression of Oct4, Sox2, Klf4, and c-Myc (OSKM) induced adult CM dedifferentiation and confers regenerative capacity upon adult hearts (Chen et al., 2021). These attempts are similar to direct reprogramming *in vivo*. Although overexpression of transcription factors seems an attractive way to promote CM proliferation, the efficiency and safety requires further investigation. Novel methods allowing precise temporal overexpression of reprogramming factors are also important.

### Intervention Using Noncoding RNAs

Only 1%–2% of genes in the mammalian genome encode proteins, and the rest are transcribed into noncoding (nc) RNAs that include micro (mi), long noncoding (lnc) and circular (circ) types. Noncoding RNAs play key roles in gene regulation during development, as well as in health and cardiovascular diseases (Poller et al., 2018). The overexpression of miR-199a, which accelerated CM entry into the cell cycle, also promoted cardiac repair, increased muscle mass, and resulted in improved cardiac systolic function in infarcted pig hearts (Eulalio et al., 2012; Gabisonia et al., 2019). The miR-302-367 cluster is expressed in the early developmental stage of mouse hearts and participates in modulating CM proliferation during the embryonic stage. Its overexpression promoted CM proliferation, probably by inhibiting the Hippo signaling pathway and reducing the size of scars after myocardial infarction (Barroso-del Jesus et al., 2008; Tian et al., 2015). MicroRNA-128 suppressed expression of the chromatin modifier SUZ12, which suppresses the CDK inhibitor p27, and activated cyclin E and CDK2. The proliferation period of neonatal mouse CMs was prolonged by miR-128 KO, and scar size and cardiac function after myocardial infarction was improved in adult miR-128 KO mice (Huang et al., 2018). MicroRNA-294 is expressed during prenatal development, but not in adult CMs. The ectopic transient expression of miR-294 recapitulated developmental signaling in CMs and promoted cell-cycle re-entry, which leads to improved cardiac function after myocardial infarction in mice (Borden et al., 2019). Long noncoding RNAs (>200 nt in length) play crucial roles in heart regeneration by promoting the proliferation of extant CMs (Han and Yang, 2021). Long noncoding RNAs associated with CM proliferation include cardiomyocyte regeneration-related lncRNA (CRRL) (Chen et al., 2018), cardiac regeneration-related lncRNA (CAREL) (Cai et al., 2018), CM proliferation regulator (CPR) (Ponnusamy et al., 2019), and endogenous cardiac regeneration-associated regulator (ECRAR) (Chen et al., 2019). Circular RNAs are lncRNAs that are characterized by a covalently closed loop structure, stable expression, and resistance to nucleases due to not having 3′ poly(A) tails and 5′ cap structures (Mester-Tonczar et al., 2020). The expression of most known circRNAs is cell-and tissue-specific and they can interact with RNA-binding proteins to form complexes that promote the expression of downstream targets. For example, circFndc3b modulates cardiac repair after myocardial infarction by interacting with the RNA-Binding Protein Fused In Sarcoma (FUS) to promote Vascular Endothelial Growth Factor (VEGF) expression (Garikipati et al., 2019) in endothelial cells. In addition, circRNA, as a miRNA sponge, regulates the expression of its downstream molecules, ultimately activating or inactivating relevant signaling pathways and generating corresponding biological functions (Hansen et al., 2013). Silencing circRNA Homeodomain-Interacting Protein Kinase 3 (HIPK3), which sponges nine miRNAs with 18 potential binding sites, significantly inhibited human cell growth (Zheng et al., 2016). Expression of the circHIPK3 is relatively abundant in the fetal and neonatal murine CM, and knockdown or overexpression of circHIPK3 respectively inhibited and increased CM proliferation *in vitro* and *in vivo* (Si et al., 2020).

### Intervention *via* Regulation of Signaling Pathways

Hippo, Notch, HIF-1α-regulated cell stress, and NRG1/ErbB4 are among the numerous signaling pathways that are involved in CM proliferation (Zhu Y. et al., 2021).

The Hippo pathway plays a pivotal role in determining heart size by regulating CM proliferation, differentiation, and apoptosis (Xin et al., 2013; Tian et al., 2015; Ikeda et al., 2019). The activation of this pathway leads to phosphorylation of the transcriptional co-activators Yes-associated protein (YAP) and Tafazzin (Taz) and the prevention of cell proliferation and regeneration. Inhibited Hippo signaling during heart development promotes CM proliferation and leads to cardiomegaly (Heallen et al., 2011). Deletion of the Hippo pathway component Salv in murine hearts after myocardial infarction induced a reparative genetic program with increased scar border vascularity, reduced fibrosis, and reversed systolic function (Leach et al., 2017). Activation of YAP activity by either an AAV9 vector or a small molecule after myocardial infarction enhanced CM proliferation and improved heart function (Lin et al., 2014; Hara et al., 2018). The extracellular matrix protein Agrin, which is expressed mainly in endothelial cells, promoted the division of CMs partially through Yap-mediated signaling (Bassat et al., 2017). Agrin is also essential for epicardial epithelial-to-mesenchymal transition during heart development (Sun et al., 2021), suggesting the importance of extracellular matrix for the regulation of cellular plasticity and proliferation.

The Notch pathway controls cellular proliferation and trabeculation in developing hearts by activating bone morphogenetic protein BMP10, which increases CM entry into the cell cycle by downregulating cell-cycle inhibitors p21, p27, and p75 (Chen et al., 2004; Grego-Bessa et al., 2007). Other reports using zebrafish after ventricular resection revealed that Notch receptors are upregulated in endocardium and epicardium, but not in CMs. Endocardial Notch signaling increases the expression of secreted Wnt antagonists (for example, Wif1 and Notum1b) and thereby suppresses hyperactivation of Wnt signaling to support CM proliferation (Zhao et al., 2014, 2019).

Hypoxia-related cell stress signaling, which is mainly regulated by HIF-1α, plays an important role in CM cell-cycle arrest and proliferation. A metabolic switch in the perinatal heart from anaerobic to aerobic is associated with the exit of CMs from the cell cycle and reduced mitotic potential. Postnatal hypoxemia prolongs, whereas hyperoxemia narrows the postnatal proliferative window of CMs (Puente et al., 2014). A rare population of hypoxic CMs can promote cell proliferation *via* HIF-1α-regulated cell stress signaling (Guimarães-Camboa et al., 2015), and hypoxic CM populations contribute to new CM formation (Kimura et al., 2015). In addition, the regenerative response of cardiomyocytes in mice exposed to gradual systemic hypoxemia 1 week after the induction of myocardial infarction become robust, resulting in decreased myocardial fibrosis and improvement of left ventricular function (Nakada et al., 2017).

Neuregulin 1 (NRG1) belongs to the epidermal growth factor gene family. It is expressed in endothelial cells and exerts biological functions *via* the ErbB family of tyrosine kinase receptors. NRG1/Erb signaling promotes CM proliferation and is a promising candidate for restoring cardiac function after injury. Activating either NRG1 (Bersell et al., 2009; Cohen et al., 2014; Polizzotti et al., 2015) or ErbB2/4 (Bersell et al., 2009; Belmonte et al., 2015; D'Uva et al., 2015) improved CM proliferation, suggesting that the NRG1/Erb signaling pathway is conserved and thus might serve as an effective therapeutic strategy for heart diseases. Recombinant human NRG1 is currently being evaluated in clinical trials of agents to treat chronic heart failure.

Other signaling pathways, such as Wnt/β-catenin (Kerkela et al., 2008; Fan et al., 2018) and Jak/Stat (Fang et al., 2013; Miyawaki et al., 2017) also stimulate CM cell-cycle re-entry. A recent report using a cocktail of five small molecules which increase CM proliferation revealed lactate-LacRS2-mediated signaling as a novel mechanism for cardiomyocyte cell-cycle re-entry (Du et al., 2022). These multiple signaling pathways within CMs crosstalk and stimulate the proliferation of extant CMs (Lin et al., 2015). Therefore, manipulating several cell cycle-related signaling pathways will be a promising strategy with which to stimulate CM proliferation.

### The Effect of Endocardial and Epicardial Factors on Cardiac Regeneration

As mentioned above, several important factors (for example, Notch, Agrin, and NRG1) to stimulate CM proliferation are expressed in non-CMs. Endocardium and epicardium are especially important in CM regeneration. The production of retinoic acid in the endocardium and epicardium is necessary for zebrafish heart regeneration and CM proliferation (Kikuchi et al., 2011). Fibronectin, which is secreted from the epicardium, can indirectly affect the process of cardiac regeneration near the injury site (Wang et al., 2013). Hand2, one of the important factors for direct reprogramming, is also expressed in the epicardium in addition to the myocardium during cardiac injury and impacts cardiac regeneration (Schindler et al., 2014). In adult mammalian heart, epicardial (but not myocardial) follistatin-like1 (Fstl1) can promote cell cycle re-entry and division of pre-existing CMs, thereby improving cardiac function and survival in murine and swine models of myocardial infarction (Wei et al., 2015). In addition to CM proliferation, revascularization after cardiac injury is regulated by epicardial Cxcl12/Cxcr4 and endocardial Vegfa signaling (Marín-Juez et al., 2019). All of these reports indicate that endocardium and epicardium play a crucial role for cardiac regeneration in response to cardiac injury.

## Conclusion

CMs usually lose their proliferative capacity in adult mammals. This largely limits heart regeneration after injury, which leads to adverse cardiac remodeling and heart failure. The mechanisms and the possibility of heart regeneration have been investigated for decades. In this review, we summarized the major approaches to manipulate cardiomyocyte plasticity for heart regeneration, namely iPSC-CM induction, direct reprogramming cardiac fibroblasts into CMs, and using molecules and signaling pathways to promote cell-cycle re-entry and the mitosis of extant CMs. All three strategies provide hope for heart regeneration and functional recovery in the clinical setting. However, their clinical applications have several limitations. For example, persistent and uncontrolled expression of miR-199a in pigs resulted in myocardial infiltration by proliferating CMs with a poorly differentiated myoblastic phenotype that leads to sudden arrhythmic death (Gabisonia et al., 2019). Similarly, although Notch and Wnt signaling is known to augment CM proliferation, their hyperactivation was shown to contrarily suppress heart regeneration (Zhao et al., 2014, 2019). Therefore, precise cardiac targeting for the delivery of therapeutics, as well as exquisite manipulation of temporally overexpressed target genes, would be of great importance. For the interventions to stimulate CM proliferation, a combination of genetic perturbation and single-cell analysis will be a powerful tool to identify novel therapeutic targets and also useful to identify optimal combinations of multiple target genes. Chemical compounds which regulate the key signaling pathways mentioned in this review will also be alternative choices. Since the technology of high-throughput drug screening has made great strides in recent years by the contribution of computer science such as machine learning and deep learning, novel drugs that produce robust proliferation in CMs may be discovered in the near future. Multidisciplinary cooperation is also needed for a more comprehensive and systematic understanding of cardiomyocyte plasticity to improve the safety and efficiency of heart regeneration. The future promises an important breakthrough in this field when various concerns and technical restrictions regarding heart regeneration are overcome.

## References

[B1] Abouleisa R. R. E. Salama A. B. M. Ou Q. Tang X.-L. Solanki M. Guo Y. (2022). Transient Cell Cycle Induction in Cardiomyocytes to Treat Subacute Ischemic Heart Failure. Circulation 145, 1339–1355. *Circulation* in press. 10.1161/circulationaha.121.057641 35061545 PMC9038650

[B2] Addis R. C. Ifkovits J. L. Pinto F. Kellam L. D. Esteso P. Rentschler S. (2013). Optimization of Direct Fibroblast Reprogramming to Cardiomyocytes Using Calcium Activity as a Functional Measure of Success. J. Mol. Cell. Cardiol. 60, 97–106. 10.1016/j.yjmcc.2013.04.004 23591016 PMC3679282

[B3] Barroso-delJesus A. Romero-López C. Lucena-Aguilar G. Melen G. J. Sanchez L. Ligero G. (2008). Embryonic Stem Cell-Specific miR302-367 Cluster: Human Gene Structure and Functional Characterization of its Core Promoter. Mol. Cell. Biol. 28, 6609–6619. 10.1128/mcb.00398-08 18725401 PMC2573233

[B4] Bassat E. Mutlak Y. E. Genzelinakh A. Shadrin I. Y. Baruch Umansky K. Yifa O. (2017). The Extracellular Matrix Protein Agrin Promotes Heart Regeneration in Mice. Nature 547, 179–184. 10.1038/nature22978 28581497 PMC5769930

[B5] Belmonte F. Das S. Sysa-Shah P. Sivakumaran V. Stanley B. Guo X. (2015). ErbB2 Overexpression Upregulates Antioxidant Enzymes, Reduces Basal Levels of Reactive Oxygen Species, and Protects Against Doxorubicin Cardiotoxicity. Am. J. Physiology-Heart Circulatory Physiology 309, H1271–H1280. 10.1152/ajpheart.00517.2014 PMC4666964 26254336

[B6] Bersell K. Arab S. Haring B. Kühn B. (2009). Neuregulin1/ErbB4 Signaling Induces Cardiomyocyte Proliferation and Repair of Heart Injury. Cell 138, 257–270. 10.1016/j.cell.2009.04.060 19632177

[B7] Borden A. Kurian J. Nickoloff E. Yang Y. Troupes C. D. Ibetti J. (2019). Transient Introduction of miR-294 in the Heart Promotes Cardiomyocyte Cell Cycle Reentry After Injury. Circ. Res. 125, 14–25. 10.1161/CIRCRESAHA.118.314223 30964391 PMC6586499

[B8] Burridge P. W. Matsa E. Shukla P. Lin Z. C. Churko J. M. Ebert A. D. (2014). Chemically Defined Generation of Human Cardiomyocytes. Nat. Methods 11, 855–860. 10.1038/nMeth.2999 24930130 PMC4169698

[B9] Cai B. Ma W. Ding F. Zhang L. Huang Q. Wang X. (2018). The Long Noncoding RNA CAREL Controls Cardiac Regeneration. J. Am. Coll. Cardiol. 72, 534–550. 10.1016/j.jacc.2018.04.085 30056829

[B10] Cao N. Huang Y. Zheng J. Spencer C. I. Zhang Y. Fu J.-D. (2016). Conversion of Human Fibroblasts into Functional Cardiomyocytes by Small Molecules. Science 352, 1216–1220. 10.1126/science.aaf1502 27127239

[B11] Chakraborty S. Sengupta A. Yutzey K. E. (2013). Tbx20 Promotes Cardiomyocyte Proliferation and Persistence of Fetal Characteristics in Adult Mouse Hearts. J. Mol. Cell. Cardiol. 62, 203–213. 10.1016/j.yjmcc.2013.05.018 23751911

[B12] Chambers D. C. Perch M. Zuckermann A. Cherikh W. S. Harhay M. O. Hayes D. (2021). The International Thoracic Organ Transplant Registry of the International Society for Heart and Lung Transplantation: Thirty-Eighth Adult Lung Transplantation Report - 2021; Focus on Recipient Characteristics. J. Heart Lung Transplant. 40, 1060–1072. 10.1016/j.healun.2021.07.021 34446355 PMC10285650

[B13] Chang Y. Lee E. Kim J. Kwon Y.-W. Kwon Y. Kim J. (2019192). Efficient *In Vivo* Direct Conversion of Fibroblasts into Cardiomyocytes Using a Nanoparticle-Based Gene Carrier. Biomaterials 192, 500–509. 10.1016/j.biomaterials.2018.11.034 30513475

[B14] Chen G. Li H. Li X. Li B. Zhong L. Huang S. (2018). Loss of Long Non-Coding RNA CRRL Promotes Cardiomyocyte Regeneration and Improves Cardiac Repair by Functioning as a Competing Endogenous RNA. J. Mol. Cell. Cardiol. 122, 152–164. 10.1016/j.yjmcc.2018.08.013 30125571

[B15] Chen H. Shi S. Acosta L. Li W. Lu J. Bao S. (2004). BMP10 Is Essential for Maintaining Cardiac Growth During Murine Cardiogenesis. Development 131, 2219–2231. 10.1242/dev.01094 15073151 PMC2628765

[B16] Chen Y. Li X. Li B. Wang H. Li M. Huang S. (2019). Long Non-Coding RNA ECRAR Triggers Post-Natal Myocardial Regeneration by Activating ERK1/2 Signaling. Mol. Ther. 27, 29–45. 10.1016/j.ymthe.2018.10.021 30528086 PMC6319349

[B17] Chen Y. Lüttmann F. F. Schoger E. Schöler H. R. Zelarayán L. C. Kim K.-P. (2021). Reversible Reprogramming of Cardiomyocytes to a Fetal State Drives Heart Regeneration in Mice. Science 80-373, 1537–1540. 10.1126/science.abg5159 34554778

[B18] Cheng R. K. Asai T. Tang H. Dashoush N. H. Kara R. J. Costa K. D. (2007). Cyclin A2 Induces Cardiac Regeneration After Myocardial Infarction and Prevents Heart Failure. Circulation Res. 100, 1741–1748. 10.1161/CIRCRESAHA.107.153544 17495221

[B19] Chow A. Stuckey D. J. Kidher E. Rocco M. Jabbour R. J. Mansfield C. A. (2017). Human Induced Pluripotent Stem Cell-Derived Cardiomyocyte Encapsulating Bioactive Hydrogels Improve Rat Heart Function Post Myocardial Infarction. Stem Cell Rep. 9, 1415–1422. 10.1016/j.stemcr.2017.09.003 PMC5830963 28988988

[B20] Christoforou N. Chakraborty S. Kirkton R. D. Adler A. F. Addis R. C. Leong K. W. (2017). Core Transcription Factors, MicroRNAs, and Small Molecules Drive Transdifferentiation of Human Fibroblasts Towards the Cardiac Cell Lineage. Sci. Rep. 7, 1–15. 10.1038/srep40285 28071742 PMC5223186

[B21] Cohen J. E. Purcell B. P. MacArthur J. W. Mu A. Shudo Y. Patel J. B. (2014). A Bioengineered Hydrogel System Enables Targeted and Sustained Intramyocardial Delivery of Neuregulin, Activating the Cardiomyocyte Cell Cycle and Enhancing Ventricular Function in a Murine Model of Ischemic Cardiomyopathy. Circ. Heart Fail. 7, 619–626. 10.1161/CIRCHEARTFAILURE.113.001273 24902740 PMC4157671

[B22] Dal-Pra S. Hodgkinson C. P. Mirotsou M. Kirste I. Dzau V. J. (2017). Demethylation of H3K27 Is Essential for the Induction of Direct Cardiac Reprogramming by MIR Combo. Circ. Res. 120, 1403–1413. 10.1161/CIRCRESAHA.116.308741 28209718 PMC5409871

[B23] Di Stefano V. Giacca M. Capogrossi M. C. Crescenzi M. Martelli F. (2011). Knockdown of Cyclin-Dependent Kinase Inhibitors Induces Cardiomyocyte Re-Entry in the Cell Cycle. J. Biol. Chem. 286, 8644–8654. 10.1074/jbc.M110.184549 21209082 PMC3048746

[B24] Du J. Zheng L. Gao P. Yang H. Yang W.-J. Guo F. (2022). A Small-Molecule Cocktail Promotes Mammalian Cardiomyocyte Proliferation and Heart Regeneration. Cell Stem Cell 29, 545–558.e13. 10.1016/j.stem.2022.03.009 35395187

[B25] D’Uva G. Aharonov A. Lauriola M. Kain D. Yahalom-Ronen Y. Carvalho S. (2015). ERBB2 Triggers Mammalian Heart Regeneration by Promoting Cardiomyocyte Dedifferentiation and Proliferation. Nat. Cell Biol. 17, 627–638. 10.1038/ncb3149 25848746

[B26] Ebelt H. Hufnagel N. Neuhaus P. Neuhaus H. Gajawada P. Simm A. (2005). Divergent Siblings. Circulation Res. 96, 509–517. 10.1161/01.RES.0000159705.17322.57 15718499

[B27] Ebelt H. Zhang Y. Kampke A. Xu J. Schlitt A. Buerke M. (2008). E2F2 Expression Induces Proliferation of Terminally Differentiated Cardiomyocytes *In Vivo* . Cardiovasc. Res. 80, 219–226. 10.1093/cvr/cvn194 18628254

[B28] Ebert A. Joshi A. U. Andorf S. Dai Y. Sampathkumar S. Chen H. (2019). Proteasome-Dependent Regulation of Distinct Metabolic States During Long-Term Culture of Human iPSC-Derived Cardiomyocytes. Circ. Res. 125, 90–103. 10.1161/CIRCRESAHA.118.313973 31104567 PMC6613799

[B29] Eulalio A. Mano M. Ferro M. D. Zentilin L. Sinagra G. Zacchigna S. (2012). Functional Screening Identifies miRNAs Inducing Cardiac Regeneration. Nature 492, 376–381. 10.1038/nature11739 23222520

[B30] Fan Y. Ho B. X. Pang J. K. S. Pek N. M. Q. Hor J. H. Ng S.-Y. (2018). Wnt/β-Catenin-Mediated Signaling Re-Activates Proliferation of Matured Cardiomyocytes. Stem Cell Res. Ther. 9, 338. 10.1186/s13287-018-1086-8 30526659 PMC6286613

[B31] Fang Y. Gupta V. Karra R. Holdway J. E. Kikuchi K. Poss K. D. (2013). Translational Profiling of Cardiomyocytes Identifies an Early Jak1/Stat3 Injury Response Required for Zebrafish Heart Regeneration. Proc. Natl. Acad. Sci. U.S.A. 110, 13416–13421. 10.1073/pnas.1309810110 23901114 PMC3746860

[B32] Fu J.-D. Stone N. R. Liu L. Spencer C. I. Qian L. Hayashi Y. (2013). Direct Reprogramming of Human Fibroblasts Toward a Cardiomyocyte-Like State. Stem Cell Rep. 1, 235–247. 10.1016/j.stemcr.2013.07.005 PMC3849259 24319660

[B33] Fu X. Khalil H. Kanisicak O. Boyer J. G. Vagnozzi R. J. Maliken B. D. (2018). Specialized Fibroblast Differentiated States Underlie Scar Formation in the Infarcted Mouse Heart. J. Clin. Investig. 128, 2127–2143. 10.1172/JCI98215 29664017 PMC5957472

[B34] Fu Y. Huang C. Xu X. Gu H. Ye Y. Jiang C. (2015). Direct Reprogramming of Mouse Fibroblasts into Cardiomyocytes with Chemical Cocktails. Cell Res. 25, 1013–1024. 10.1038/cr.2015.99 26292833 PMC4559819

[B35] Funakoshi S. Fernandes I. Mastikhina O. Wilkinson D. Tran T. Dhahri W. (2021). Generation of Mature Compact Ventricular Cardiomyocytes from Human Pluripotent Stem Cells. Nat. Commun. 12. 10.1038/s41467-021-23329-z PMC8155185 34039977

[B36] Gabisonia K. Prosdocimo G. Aquaro G. D. Carlucci L. Zentilin L. Secco I. (2019). MicroRNA Therapy Stimulates Uncontrolled Cardiac Repair After Myocardial Infarction in Pigs. Nature 569, 418–422. 10.1038/s41586-019-1191-6 31068698 PMC6768803

[B37] Gao L. Gregorich Z. R. Zhu W. Mattapally S. Oduk Y. Lou X. (2018). Large Cardiac Muscle Patches Engineered from Human Induced-Pluripotent Stem Cell-Derived Cardiac Cells Improve Recovery from Myocardial Infarction in Swine. Circulation 137, 1712–1730. 10.1161/circulationaha.117.030785 29233823 PMC5903991

[B38] Gao L. Kupfer M. E. Jung J. P. Yang L. Zhang P. Da Sie Y. (2017). Myocardial Tissue Engineering with Cells Derived from Human-Induced Pluripotent Stem Cells and a Native-Like, High-Resolution, 3-Dimensionally Printed Scaffold. Circ. Res. 120, 1318–1325. 10.1161/circresaha.116.310277 28069694 PMC5392171

[B39] Garbern J. C. Helman A. Sereda R. Sarikhani M. Ahmed A. Escalante G. O. (2020). Inhibition of mTOR Signaling Enhances Maturation of Cardiomyocytes Derived from Human-Induced Pluripotent Stem Cells via P53-Induced Quiescence. Circulation 141, 285–300. 10.1161/circulationaha.119.044205 31707831 PMC7009740

[B40] Garikipati V. N. S. Verma S. K. Cheng Z. Liang D. Truongcao M. M. Cimini M. (2019). Circular RNA CircFndc3b Modulates Cardiac Repair After Myocardial Infarction via FUS/VEGF-A Axis. Nat. Commun. 10, 4317. 10.1038/s41467-019-11777-7 31541092 PMC6754461

[B41] Garry G. A. Bezprozvannaya S. Chen K. Zhou H. Hashimoto H. Morales M. G. (2021). The Histone Reader PHF7 Cooperates with the SWI/SNF Complex at Cardiac Super Enhancers to Promote Direct Reprogramming. Nat. Cell Biol. 23, 467–475. 10.1038/s41556-021-00668-z 33941892 PMC8243412

[B42] Gentillon C. Li D. Duan M. Yu W.-M. Preininger M. K. Jha R. (2019). Targeting HIF-1α in Combination with PPARα Activation and Postnatal Factors Promotes the Metabolic Maturation of Human Induced Pluripotent Stem Cell-Derived Cardiomyocytes. J. Mol. Cell. Cardiol. 132, 120–135. 10.1016/j.yjmcc.2019.05.003 31082397 PMC6683286

[B43] Gomez-Garcia M. J. Quesnel E. Al-attar R. Laskary A. R. Laflamme M. A. (2021). Maturation of Human Pluripotent Stem Cell Derived Cardiomyocytes *In Vitro* and *In Vivo* . Seminars Cell & Dev. Biol. 118, 163–171. 10.1016/j.semcdb.2021.05.022 34053865

[B44] Grego-Bessa J. Luna-Zurita L. del Monte G. Bolós V. Melgar P. Arandilla A. (2007). Notch Signaling Is Essential for Ventricular Chamber Development. Dev. Cell 12, 415–429. 10.1016/j.devcel.2006.12.011 17336907 PMC2746361

[B45] Guimarães-Camboa N. Stowe J. Aneas I. Sakabe N. Cattaneo P. Henderson L. (2015). HIF1α Represses Cell Stress Pathways to Allow Proliferation of Hypoxic Fetal Cardiomyocytes. Dev. Cell 33, 507–521. 10.1016/j.devcel.2015.04.021 26028220 PMC4509618

[B46] Haginiwa S. Sadahiro T. Kojima H. Isomi M. Tamura F. Kurotsu S. (2019). Tbx6 Induces Cardiomyocyte Proliferation in Postnatal and Adult Mouse Hearts. Biochem. Biophysical Res. Commun. 513, 1041–1047. 10.1016/j.bbrc.2019.04.087 31010673

[B47] Han L. Yang L. (2021). Multidimensional Mechanistic Spectrum of Long Non-Coding RNAs in Heart Development and Disease. Front. Cardiovasc. Med. 8, 1–12. 10.3389/fcvm.2021.728746 PMC8483262 34604357

[B48] Hansen T. B. Jensen T. I. Clausen B. H. Bramsen J. B. Finsen B. Damgaard C. K. (2013). Natural RNA Circles Function as Efficient microRNA Sponges. Nature 495, 384–388. 10.1038/nature11993 23446346

[B49] Hara H. Takeda N. Kondo M. Kubota M. Saito T. Maruyama J. (2018). Discovery of a Small Molecule to Increase Cardiomyocytes and Protect the Heart After Ischemic Injury. JACC Basic Transl. Sci. 3, 639–653. 10.1016/j.jacbts.2018.07.005 30456335 PMC6234526

[B50] Hashimoto H. Wang Z. Garry G. A. Malladi V. S. Botten G. A. Ye W. (2019). Cardiac Reprogramming Factors Synergistically Activate Genome-Wide Cardiogenic Stage-Specific Enhancers. Cell Stem Cell 25, 69–86. e5. 10.1016/j.stem.2019.03.022 31080136 PMC6754266

[B51] Hatzistergos K. E. Williams A. R. Dykxhoorn D. Bellio M. A. Yu W. Hare J. M. (2019). Tumor Suppressors RB1 and CDKN2a Cooperatively Regulate Cell-Cycle Progression and Differentiation During Cardiomyocyte Development and Repair. Circ. Res. 124, 1184–1197. 10.1161/circresaha.118.314063 30744497

[B52] Heallen T. Zhang M. Wang J. Bonilla-Claudio M. Klysik E. Johnson R. L. (2011). Hippo Pathway Inhibits Wnt Signaling to Restrain Cardiomyocyte Proliferation and Heart Size. Science 332, 458–461. 10.1126/science.1199010 21512031 PMC3133743

[B53] Hirai H. Kikyo N. (2014). Inhibitors of Suppressive Histone Modification Promote Direct Reprogramming of Fibroblasts to Cardiomyocyte-Like Cells. Cardiovasc. Res. 102, 188–190. 10.1093/cvr/cvu023 24477643 PMC3958621

[B54] Hou P. Li Y. Zhang X. Liu C. Guan J. Li H. (2013). Pluripotent Stem Cells Induced from Mouse Somatic Cells by Small-Molecule Compounds. Science 341, 651–654. 10.1126/science.1239278 23868920

[B55] Hu D. Linders A. Yamak A. Correia C. Kijlstra J. D. Garakani A. (2018). Metabolic Maturation of Human Pluripotent Stem Cell-Derived Cardiomyocytes by Inhibition of HIF1α and LDHA. Circ. Res. 123, 1066–1079. 10.1161/CIRCRESAHA.118.313249 30355156 PMC6208155

[B56] Huang W. Feng Y. Liang J. Yu H. Wang C. Wang B. (2018). Loss of microRNA-128 Promotes Cardiomyocyte Proliferation and Heart Regeneration. Nat. Commun. 9, 700. 10.1038/s41467-018-03019-z 29453456 PMC5816015

[B57] Ieda M. Fu J.-D. Delgado-Olguin P. Vedantham V. Hayashi Y. Bruneau B. G. (2010). Direct Reprogramming of Fibroblasts into Functional Cardiomyocytes by Defined Factors. Cell 142, 375–386. 10.1016/j.cell.2010.07.002 20691899 PMC2919844

[B58] Ikeda S. Mizushima W. Sciarretta S. Abdellatif M. Zhai P. Mukai R. (2019). Hippo Deficiency Leads to Cardiac Dysfunction Accompanied by Cardiomyocyte Dedifferentiation During Pressure Overload. Circ. Res. 124, 292–305. 10.1161/CIRCRESAHA.118.314048 30582455 PMC6645688

[B59] Inagawa K. Miyamoto K. Yamakawa H. Muraoka N. Sadahiro T. Umei T. (2012). Induction of Cardiomyocyte-Like Cells in Infarct Hearts by Gene Transfer of Gata4, Mef2c, and Tbx5. Circ. Res. 111, 1147–1156. 10.1161/CIRCRESAHA.112.271148 22931955

[B60] Isomi M. Sadahiro T. Yamakawa H. Fujita R. Yamada Y. Abe Y. (2021). Overexpression of Gata4, Mef2c, and Tbx5 Generates Induced Cardiomyocytes via Direct Reprogramming and Rare Fusion in the Heart. Circulation 143, 2123–2125. 10.1161/CIRCULATIONAHA.120.052799 34029137

[B61] Ito E. Miyagawa S. Takeda M. Kawamura A. Harada A. Iseoka H. (2019). Tumorigenicity Assay Essential for Facilitating Safety Studies of hiPSC-Derived Cardiomyocytes for Clinical Application. Sci. Rep. 9, 1–10. 10.1038/s41598-018-38325-5 30760836 PMC6374479

[B62] Jayawardena T. M. Egemnazarov B. Finch E. A. Zhang L. Payne J. A. Pandya K. (2012). MicroRNA-Mediated *In Vitro* and *In Vivo* Direct Reprogramming of Cardiac Fibroblasts to Cardiomyocytes. Circ. Res. 110, 1465–1473. 10.1161/CIRCRESAHA.112.269035 22539765 PMC3380624

[B63] Jayawardena T. M. Finch E. A. Zhang L. Zhang H. Hodgkinson C. P. Pratt R. E. (2015). MicroRNA Induced Cardiac Reprogramming *In Vivo* . Circ. Res. 116, 418–424. 10.1161/CIRCRESAHA.116.304510 25351576 PMC4312531

[B64] Jenča D. Melenovský V. Stehlik J. Staněk V. Kettner J. Kautzner J. (2021). Heart Failure After Myocardial Infarction: Incidence and Predictors. Esc. Heart Fail. 8, 222–237. 10.1002/ehf2.13144 33319509 PMC7835562

[B65] Kawaguchi S. Soma Y. Nakajima K. Kanazawa H. Tohyama S. Tabei R. (2021). Intramyocardial Transplantation of Human iPS Cell-Derived Cardiac Spheroids Improves Cardiac Function in Heart Failure Animals. JACC Basic Transl. Sci. 6, 239–254. 10.1016/j.jacbts.2020.11.017 33778211 PMC7987543

[B66] Kawai Y. Tohyama S. Arai K. Tamura T. Soma Y. Fukuda K. (2022). Scaffold-Free Tubular Engineered Heart Tissue from Human Induced Pluripotent Stem Cells Using Bio-3D Printing Technology *In Vivo* . Front. Cardiovasc. Med. 8, 1–11. 10.3389/fcvm.2021.806215 PMC8811174 35127867

[B67] Kawamura M. Miyagawa S. Fukushima S. Saito A. Miki K. Funakoshi S. (2017). Enhanced Therapeutic Effects of Human iPS Cell Derived-Cardiomyocyte by Combined Cell-Sheets with Omental Flap Technique in Porcine Ischemic Cardiomyopathy Model. Sci. Rep. 7, 1–11. 10.1038/s41598-017-08869-z 28821761 PMC5562896

[B68] Kerkela R. Kockeritz L. MacAulay K. Zhou J. Doble B. W. Beahm C. (2008). Deletion of GSK-3β in Mice Leads to Hypertrophic Cardiomyopathy Secondary to Cardiomyoblast Hyperproliferation. J. Clin. Investig. 118, 3609–3618. 10.1172/JCI36245 18830417 PMC2556242

[B69] Kikuchi K. Holdway J. E. Major R. J. Blum N. Dahn R. D. Begemann G. (2011). Retinoic Acid Production by Endocardium and Epicardium Is an Injury Response Essential for Zebrafish Heart Regeneration. Dev. Cell 20, 397–404. 10.1016/j.devcel.2011.01.010 21397850 PMC3071981

[B70] Kimura W. Xiao F. Canseco D. C. Muralidhar S. Thet S. Zhang H. M. (2015). Hypoxia Fate Mapping Identifies Cycling Cardiomyocytes in the Adult Heart. Nature 523, 226–230. 10.1038/nature14582 26098368

[B71] Kuppusamy K. T. Jones D. C. Sperber H. Madan A. Fischer K. A. Rodriguez M. L. (2015). Let-7 Family of microRNA Is Required for Maturation and Adult-Like Metabolism in Stem Cell-Derived Cardiomyocytes. Proc. Natl. Acad. Sci. U.S.A. 112, E2785–E2794. 10.1073/pnas.1424042112 25964336 PMC4450404

[B72] Kurotsu S. Sadahiro T. Fujita R. Tani H. Yamakawa H. Tamura F. (2020). Soft Matrix Promotes Cardiac Reprogramming via Inhibition of YAP/TAZ and Suppression of Fibroblast Signatures. Stem Cell Rep. 15, 612–628. 10.1016/j.stemcr.2020.07.022 PMC7486305 32857980

[B73] Lam N. T. Sadek H. A. (2018). Neonatal Heart Regeneration. Circulation 138, 412–423. 10.1161/CIRCULATIONAHA.118.033648 30571359 PMC6673675

[B74] Lázár E. Sadek H. A. Bergmann O. (2017). Cardiomyocyte Renewal in the Human Heart: Insights from the Fall-Out. Eur. Heart J. 38, 2333–2342. 10.1093/eurheartj/ehx343 28810672 PMC5837331

[B75] Le Bras A. (2018). Dynamics of Fibroblast Activation in the Infarcted Heart. Nat. Rev. Cardiol. 15, 379. 10.1038/s41569-018-0025-9 29713010

[B76] Leach J. P. Heallen T. Zhang M. Rahmani M. Morikawa Y. Hill M. C. (2017). Hippo Pathway Deficiency Reverses Systolic Heart Failure After Infarction. Nature 550, 260–264. 10.1038/nature24045 28976966 PMC5729743

[B77] Lian X. Hsiao C. Wilson G. Zhu K. Hazeltine L. B. Azarin S. M. (2012). Robust Cardiomyocyte Differentiation from Human Pluripotent Stem Cells via Temporal Modulation of Canonical Wnt Signaling. Proc. Natl. Acad. Sci. U.S.A. 109. 10.1073/pnas.1200250109 PMC3390875 22645348

[B78] Lin Z. Von Gise A. Zhou P. Gu F. Ma Q. Jiang J. (2014). Cardiac-Specific YAP Activation Improves Cardiac Function and Survival in an Experimental Murine MI Model. Circ. Res. 115, 354–363. 10.1161/CIRCRESAHA.115.303632 24833660 PMC4104149

[B79] Lin Z. Zhou P. Von Gise A. Gu F. Ma Q. Chen J. (2015). Pi3kcb Links Hippo-YAP and PI3K-AKT Signaling Pathways to Promote Cardiomyocyte Proliferation and Survival. Circ. Res. 116, 35–45. 10.1161/CIRCRESAHA.115.304457 25249570 PMC4282610

[B80] Lindgren I. M. Drake R. R. Chattergoon N. N. Thornburg K. L. (2019). Down‐Regulation of MEIS1 Promotes the Maturation of Oxidative Phosphorylation in Perinatal Cardiomyocytes. FASEB J. 33, 7417–7426. 10.1096/fj.201801330RR 30884246 PMC6529342

[B81] Liu L. Guo Y. Li Z. Wang Z. (2021). Improving Cardiac Reprogramming for Heart Regeneration in Translational Medicine. Cells 10, 3297. 10.3390/cells10123297 34943805 PMC8699771

[B82] Liu Z. Chen O. Zheng M. Wang L. Zhou Y. Yin C. (2016). Re-Patterning of H3K27me3, H3K4me3 and DNA Methylation During Fibroblast Conversion into Induced Cardiomyocytes. Stem Cell Res. 16, 507–518. 10.1016/j.scr.2016.02.037 26957038 PMC4828257

[B83] Liu Z. Wang L. Welch J. D. Ma H. Zhou Y. Vaseghi H. R. (2017). Single-Cell Transcriptomics Reconstructs Fate Conversion from Fibroblast to Cardiomyocyte. Nature 551, 100–104. 10.1038/nature24454 29072293 PMC5954984

[B84] Lu F. Langenbacher A. Chen J.-N. (2017). Tbx20 Drives Cardiac Progenitor Formation and Cardiomyocyte Proliferation in Zebrafish. Dev. Biol. 421, 139–148. 10.1016/j.ydbio.2016.12.009 27940156 PMC5226859

[B85] MacLellan W. R. Garcia A. Oh H. Frenkel P. Jordan M. C. Roos K. P. (2005). Overlapping Roles of Pocket Proteins in the Myocardium Are Unmasked by Germ Line Deletion of P130 Plus Heart-Specific Deletion of Rb. Mol. Cell. Biol. 25, 2486–2497. 10.1128/mcb.25.6.2486-2497.2005 15743840 PMC1061608

[B86] Mahmoud A. I. Kocabas F. Muralidhar S. A. Kimura W. Koura A. S. Thet S. (2013). Meis1 Regulates Postnatal Cardiomyocyte Cell Cycle Arrest. Nature 497, 249–253. 10.1038/nature12054 23594737 PMC4159712

[B87] Malek Mohammadi M. Kattih B. Grund A. Froese N. Korf‐Klingebiel M. Gigina A. (2017). The Transcription Factor GATA 4 Promotes Myocardial Regeneration in Neonatal Mice. EMBO Mol. Med. 9, 265–279. 10.15252/emmm.201606602 28053183 PMC5286367

[B88] Marín-Juez R. El-Sammak H. Helker C. S. M. Kamezaki A. Mullapuli S. T. Bibli S.-I. (2019). Coronary Revascularization During Heart Regeneration Is Regulated by Epicardial and Endocardial Cues and Forms a Scaffold for Cardiomyocyte Repopulation. Dev. Cell 51, 503–515.e4. 10.1016/j.devcel.2019.10.019 31743664 PMC6982407

[B89] Meng S. Gu Q. Yang X. Lv J. Owusu I. Matrone G. (2018). TBX20 Regulates Angiogenesis Through the Prokineticin 2-Prokineticin Receptor 1 Pathway. Circulation 138, 913–928. 10.1161/CIRCULATIONAHA.118.033939 29545372 PMC6139092

[B90] Mester-Tonczar J. Hašimbegović E. Spannbauer A. Traxler D. Kastner N. Zlabinger K. (2020). Circular RNAs in Cardiac Regeneration: Cardiac Cell Proliferation, Differentiation, Survival, and Reprogramming. Front. Physiol. 11, 580465. 10.3389/fphys.2020.580465 33117197 PMC7550749

[B91] Miklas J. W. Clark E. Levy S. Detraux D. Leonard A. Beussman K. (2019). TFPa/HADHA Is Required for Fatty Acid Beta-Oxidation and Cardiolipin Re-Modeling in Human Cardiomyocytes. Nat. Commun. 10, 4671. 10.1038/s41467-019-12482-1 31604922 PMC6789043

[B92] Miyamoto K. Akiyama M. Tamura F. Isomi M. Yamakawa H. Sadahiro T. (2018). Direct *In Vivo* Reprogramming with Sendai Virus Vectors Improves Cardiac Function After Myocardial Infarction. Cell Stem Cell 22, 91–103.e5. 10.1016/j.stem.2017.11.010 29276141

[B93] Miyawaki A. Obana M. Mitsuhara Y. Orimoto A. Nakayasu Y. Yamashita T. (2017). Adult Murine Cardiomyocytes Exhibit Regenerative Activity with Cell Cycle Reentry Through STAT3 in the Healing Process of Myocarditis. Sci. Rep. 7, 1–15. 10.1038/s41598-017-01426-8 28469272 PMC5431117

[B94] Mohamed T. M. A. Ang Y.-S. Radzinsky E. Zhou P. Huang Y. Elfenbein A. (2018). Regulation of Cell Cycle to Stimulate Adult Cardiomyocyte Proliferation and Cardiac Regeneration. Cell 173, 104–116. 10.1016/j.cell.2018.02.014 29502971 PMC5973786

[B95] Mohamed T. M. A. Stone N. R. Berry E. C. Radzinsky E. Huang Y. Pratt K. (2017). Chemical Enhancement of *In Vitro* and *In Vivo* Direct Cardiac Reprogramming. Circulation 135, 978–995. 10.1161/CIRCULATIONAHA.116.024692 27834668 PMC5340593

[B96] Muraoka N. Nara K. Tamura F. Kojima H. Yamakawa H. Sadahiro T. (2019). Role of Cyclooxygenase-2-Mediated Prostaglandin E2-Prostaglandin E Receptor 4 Signaling in Cardiac Reprogramming. Nat. Commun. 10, 1–5. 10.1038/s41467-019-08626-y 30787297 PMC6382796

[B97] Muraoka N. Yamakawa H. Miyamoto K. Sadahiro T. Umei T. Isomi M. (2014). MiR‐133 Promotes Cardiac Reprogramming by Directly Repressing Snai1 and Silencing Fibroblast Signatures. EMBO J. 33, 1565–1581. 10.15252/embj.201387605 24920580 PMC4198052

[B98] Murthy N. Lee K. Yu P. Lingampalli N. Kim H. J. Tang R. (2015). Peptide-Enhanced mRNA Transfection in Cultured Mouse Cardiac Fibroblasts and Direct Reprogramming Towards Cardiomyocyte-Like Cells. Ijn 10, 1841–1854. 10.2147/IJN.S75124 25834424 PMC4358644

[B99] Nakada Y. Canseco D. C. Thet S. Abdisalaam S. Asaithamby A. Santos C. X. (2017). Hypoxia Induces Heart Regeneration in Adult Mice. Nature 541, 222–227. 10.1038/nature20173 27798600

[B100] Nakamura Y. Miyagawa S. Yoshida S. Sasawatari S. Toyofuku T. Toda K. (2019). Natural Killer Cells Impede the Engraftment of Cardiomyocytes Derived from Induced Pluripotent Stem Cells in Syngeneic Mouse Model. Sci. Rep. 9, 1–13. 10.1038/s41598-019-47134-3 31346220 PMC6658523

[B101] Nam Y.-J. Lubczyk C. Bhakta M. Zang T. Fernandez-Perez A. McAnally J. (2014). Induction of Diverse Cardiac Cell Types by Reprogramming Fibroblasts with Cardiac Transcription Factors. Dev 141, 4267–4278. 10.1242/dev.114025 PMC4302916 25344074

[B102] Nam Y.-J. Song K. Luo X. Daniel E. Lambeth K. West K. (2013). Reprogramming of Human Fibroblasts Toward a Cardiac Fate. Proc. Natl. Acad. Sci. U.S.A. 110, 5588–5593. 10.1073/pnas.1301019110 23487791 PMC3619357

[B103] Nguyen N. U. N. Canseco D. C. Xiao F. Nakada Y. Li S. Lam N. T. (2020). A Calcineurin-Hoxb13 Axis Regulates Growth Mode of Mammalian Cardiomyocytes. Nature 582, 271–276. 10.1038/s41586-020-2228-6 32499640 PMC7670845

[B104] Ogawa M. Geng F.-S. Humphreys D. T. Kristianto E. Sheng D. Z. Hui S. P. (2021). Krüppel-Like Factor 1 Is a Core Cardiomyogenic Trigger in Zebrafish. Science 372, 201–205. 10.1126/science.abe2762 33833125

[B105] Paige S. L. Thomas S. Stoick-Cooper C. L. Wang H. Maves L. Sandstrom R. (2012). A Temporal Chromatin Signature in Human Embryonic Stem Cells Identifies Regulators of Cardiac Development. Cell 151, 221–232. 10.1016/j.cell.2012.08.027 22981225 PMC3462257

[B106] Park S.-J. Kim R. Y. Park B.-W. Lee S. Choi S. W. Park J.-H. (2019). Dual Stem Cell Therapy Synergistically Improves Cardiac Function and Vascular Regeneration Following Myocardial Infarction. Nat. Commun. 10, 1–12. 10.1038/s41467-019-11091-2 31311935 PMC6635499

[B107] Polizzotti B. D. Ganapathy B. Walsh S. Choudhury S. Ammanamanchi N. Bennett D. G. (2015). Neuregulin Stimulation of Cardiomyocyte Regeneration in Mice and Human Myocardium Reveals a Therapeutic Window. Sci. Transl. Med. 7, 281ra45. 10.1126/scitranslmed.aaa5171 PMC5360874 25834111

[B108] Poller W. Dimmeler S. Heymans S. Zeller T. Haas J. Karakas M. (2018). Non-Coding RNAs in Cardiovascular Diseases: Diagnostic and Therapeutic Perspectives. Eur. Heart J. 39, 2704–2716. 10.1093/eurheartj/ehx165 28430919 PMC6454570

[B109] Ponnusamy M. Liu F. Zhang Y.-H. Li R.-B. Zhai M. Liu F. (2019). Long Noncoding RNA CPR (Cardiomyocyte Proliferation Regulator) Regulates Cardiomyocyte Proliferation and Cardiac Repair. Circulation 139, 2668–2684. 10.1161/CIRCULATIONAHA.118.035832 30832495

[B110] Protze S. I. Lee J. H. Keller G. M. (2019). Human Pluripotent Stem Cell-Derived Cardiovascular Cells: From Developmental Biology to Therapeutic Applications. Cell Stem Cell 25, 311–327. 10.1016/j.stem.2019.07.010 31491395

[B111] Puente B. N. Kimura W. Muralidhar S. A. Moon J. Amatruda J. F. Phelps K. L. (2014). The Oxygen-Rich Postnatal Environment Induces Cardiomyocyte Cell-Cycle Arrest Through DNA Damage Response. Cell 157, 565–579. 10.1016/j.cell.2014.03.032 24766806 PMC4104514

[B112] Qian L. Huang Y. Spencer C. I. Foley A. Vedantham V. Liu L. (2012). *In Vivo* reprogramming of Murine Cardiac Fibroblasts into Induced Cardiomyocytes. Nature 485, 593–598. 10.1038/nature11044 22522929 PMC3369107

[B113] Querdel E. Reinsch M. Castro L. Köse D. Bähr A. Reich S. (2021). Human Engineered Heart Tissue Patches Remuscularize the Injured Heart in a Dose-Dependent Manner. Circulation 143, 1991–2006. 10.1161/CIRCULATIONAHA.120.047904 33648345 PMC8126500

[B114] Riching A. S. Danis E. Zhao Y. Cao Y. Chi C. Bagchi R. A. (2021). Suppression of Canonical TGF-β Signaling Enables GATA4 to Interact with H3K27me3 Demethylase JMJD3 to Promote Cardiomyogenesis. J. Mol. Cell. Cardiol. 153, 44–59. 10.1016/j.yjmcc.2020.12.005 33359755 PMC8809092

[B115] Roost M. S. Slieker R. C. Bialecka M. Van Iperen L. Gomes Fernandes M. M. He N. (2017). DNA Methylation and Transcriptional Trajectories During Human Development and Reprogramming of Isogenic Pluripotent Stem Cells. Nat. Commun. 8. 10.1038/s41467-017-01077-3 PMC5640655 29030611

[B116] Salama A. B. M. Gebreil A. Mohamed T. M. A. Abouleisa R. R. E. (2021). Induced Cardiomyocyte Proliferation: A Promising Approach to Cure Heart Failure. Ijms 22, 7720. 10.3390/ijms22147720 34299340 PMC8303201

[B117] Schindler Y. L. Garske K. M. Wang J. Firulli B. A. Firulli A. B. Poss K. D. (2014). Hand2 Elevates Cardiomyocyte Production During Zebrafish Heart Development and Regeneration. Dev 141, 3112–3122. 10.1242/dev.106336 PMC4197543 25038045

[B118] Sdek P. Zhao P. Wang Y. Huang C.-j. Ko C. Y. Butler P. C. (2011). Rb and P130 Control Cell Cycle Gene Silencing to Maintain the Postmitotic Phenotype in Cardiac Myocytes. J. Cell Biol. 194, 407–423. 10.1083/jcb.201012049 21825075 PMC3153646

[B119] Shapiro S. D. Ranjan A. K. Kawase Y. Cheng R. K. Kara R. J. Bhattacharya R. (2014). Cyclin A2 Induces Cardiac Regeneration After Myocardial Infarction Through Cytokinesis of Adult Cardiomyocytes. Sci. Transl. Med. 6, 1–12. 10.1126/scitranslmed.3007668 24553388

[B120] Shen T. Aneas I. Sakabe N. Dirschinger R. J. Wang G. Smemo S. (2011). Tbx20 Regulates a Genetic Program Essential to Adult Mouse Cardiomyocyte Function. J. Clin. Investig. 121, 4640–4654. 10.1172/JCI59472 22080862 PMC3223071

[B121] Shiba Y. Gomibuchi T. Seto T. Wada Y. Ichimura H. Tanaka Y. (2016). Allogeneic Transplantation of iPS Cell-Derived Cardiomyocytes Regenerates Primate Hearts. Nature 538, 388–391. 10.1038/nature19815 27723741

[B122] Si X. Zheng H. Wei G. Li M. Li W. Wang H. (2020). circRNA Hipk3 Induces Cardiac Regeneration after Myocardial Infarction in Mice by Binding to Notch1 and miR-133a. Mol. Ther. - Nucleic Acids 21, 636–655. 10.1016/j.omtn.2020.06.024 32736292 PMC7393325

[B123] Song K. Nam Y.-J. Luo X. Qi X. Tan W. Huang G. N. (2012). Heart Repair by Reprogramming Non-Myocytes with Cardiac Transcription Factors. Nature 485, 599–604. 10.1038/nature11139 22660318 PMC3367390

[B124] Sougawa N. Miyagawa S. Fukushima S. Kawamura A. Yokoyama J. Ito E. (2018). Immunologic Targeting of CD30 Eliminates Tumourigenic Human Pluripotent Stem Cells, Allowing Safer Clinical Application of hiPSC-Based Cell Therapy. Sci. Rep. 8, 1–12. 10.1038/s41598-018-21923-8 29487310 PMC5829260

[B125] Stone N. R. Gifford C. A. Thomas R. Pratt K. J. B. Samse-Knapp K. Mohamed T. M. A. (2019). Context-Specific Transcription Factor Functions Regulate Epigenomic and Transcriptional Dynamics During Cardiac Reprogramming. Cell Stem Cell 25, 87–102.e9. 10.1016/j.stem.2019.06.012 31271750 PMC6632093

[B126] Sun X. Malandraki-Miller S. Kennedy T. Bassat E. Klaourakis K. Zhao J. (2021). The Extracellular Matrix Protein Agrin Is Essential for Epicardial Epithelial-To-Mesenchymal Transition During Heart Development. Dev 148, dev197525. 10.1242/dev.197525 PMC8172119 33969874

[B127] Takahashi K. Tanabe K. Ohnuki M. Narita M. Ichisaka T. Tomoda K. (2007). Induction of Pluripotent Stem Cells from Adult Human Fibroblasts by Defined Factors. Cell 131, 861–872. 10.1016/j.cell.2007.11.019 18035408

[B128] Tallquist M. D. Molkentin J. D. (2017). Redefining the Identity of Cardiac Fibroblasts. Nat. Rev. Cardiol. 14, 484–491. 10.1038/nrcardio.2017.57 28436487 PMC6329009

[B129] Tang Y. Zhao L. Yu X. Zhang J. Qian L. Jin J. (2021). Inhibition of EZH2 Primes the Cardiac Gene Activation via Removal of Epigenetic Repression During Human Direct Cardiac Reprogramming. Stem Cell Res. 53, 102365. 10.1016/j.scr.2021.102365 34087994 PMC8238038

[B130] Testa G. Russo M. Di Benedetto G. Barbato M. Parisi S. Pirozzi F. (2020). Bmi1 Inhibitor PTC-209 Promotes Chemically-Induced Direct Cardiac Reprogramming of Cardiac Fibroblasts into Cardiomyocytes. Sci. Rep. 10, 1–16. 10.1038/s41598-020-63992-8 32346096 PMC7189257

[B131] Tian Y. Liu Y. Wang T. Zhou N. Kong J. Chen L. (2015). A microRNA-Hippo Pathway that Promotes Cardiomyocyte Proliferation and Cardiac Regeneration in Mice. Sci. Transl. Med. 7, 1–12. 10.1126/scitranslmed.3010841 PMC6295313 25787764

[B132] Tohyama S. Fujita J. Hishiki T. Matsuura T. Hattori F. Ohno R. (2016). Glutamine Oxidation Is Indispensable for Survival of Human Pluripotent Stem Cells. Cell Metab. 23, 663–674. 10.1016/j.cmet.2016.03.001 27050306

[B133] Van Amerongen M. J. Diehl F. Novoyatleva T. Patra C. Engel F. B. (2010). E2F4 Is Required for Cardiomyocyte Proliferation. Cardiovasc. Res. 86, 92–102. 10.1093/cvr/cvp383 19955219

[B134] Van Handel B. Wang L. Ardehali R. (2022). Environmental Factors Influence Somatic Cell Reprogramming to Cardiomyocyte-Like Cells. Seminars Cell & Dev. Biol. 122, 44–49. 10.1016/j.semcdb.2021.05.028 34083115

[B135] Velayutham N. Agnew E. J. Yutzey K. E. (2019). Postnatal Cardiac Development and Regenerative Potential in Large Mammals. Pediatr. Cardiol. 40, 1345–1358. 10.1007/s00246-019-02163-7 31346664 PMC6786953

[B136] Virani S. S. Alonso A. Aparicio H. J. Benjamin E. J. Bittencourt M. S. Callaway C. W. (2021). Heart Disease and Stroke Statistics-2021 Update. Circulation 143. 10.1161/CIR.0000000000000950 PMC13036842 33501848

[B137] Vivien C. J. Hudson J. E. Porrello E. R. (2016). Evolution, Comparative Biology and Ontogeny of Vertebrate Heart Regeneration. npj Regen. Med. 1, 1–14. 10.1038/npjregenmed.2016.12 PMC5744704 29302337

[B138] Vliegen H. W. Van Der Laarse A. Cornelisse C. J. Eulderink F. (1991). Myocardial Changes in Pressure Overload-Induced Left Ventricular Hypertrophy. Eur. Heart J. 12, 488–494. 10.1093/oxfordjournals.eurheartj.a059928 1829680

[B139] Volland C. Schott P. Didié M. Männer J. Unsöld B. Toischer K. (2020). Control of p21Cip by BRCA1-Associated Protein Is Critical for Cardiomyocyte Cell Cycle Progression and Survival. Cardiovasc. Res. 116, 592–604. 10.1093/cvr/cvz177 31286143

[B140] Wada R. Muraoka N. Inagawa K. Yamakawa H. Miyamoto K. Sadahiro T. (2013). Induction of Human Cardiomyocyte-Like Cells from Fibroblasts by Defined Factors. Proc. Natl. Acad. Sci. U.S.A. 110, 12667–12672. 10.1073/pnas.1304053110 23861494 PMC3732928

[B141] Wamstad J. A. Alexander J. M. Truty R. M. Shrikumar A. Li F. Eilertson K. E. (2012). Dynamic and Coordinated Epigenetic Regulation of Developmental Transitions in the Cardiac Lineage. Cell 151, 206–220. 10.1016/j.cell.2012.07.035 22981692 PMC3462286

[B142] Wang C. Zhu K. Li J. Lai H. Wang C. Lai H. (2014). Reprogramming Fibroblasts to Pluripotency Using Arginine-Terminated Polyamidoamine Nanoparticles Based Non-Viral Gene Delivery System. Ijn 9, 5837–5847. 10.2147/IJN.S73961 25540584 PMC4270399

[B143] Wang H. Yang Y. Liu J. Qian L. (2021a). Direct Cell Reprogramming: Approaches, Mechanisms and Progress. Nat. Rev. Mol. Cell Biol. 22, 410–424. 10.1038/s41580-021-00335-z 33619373 PMC8161510

[B144] Wang J. Karra R. Dickson A. L. Poss K. D. (2013). Fibronectin Is Deposited by Injury-Activated Epicardial Cells and Is Necessary for Zebrafish Heart Regeneration. Dev. Biol. 382, 427–435. 10.1016/j.ydbio.2013.08.012 23988577 PMC3852765

[B145] Wang Q. Song Y. Chen J. Li Q. Gao J. Tan H. (2021b). Direct *In Vivo* Reprogramming with Non-Viral Sequential Targeting Nanoparticles Promotes Cardiac Regeneration. Biomaterials 276, 121028. 10.1016/j.biomaterials.2021.121028 34293701

[B146] Wei K. Serpooshan V. Hurtado C. Diez-Cuñado M. Zhao M. Maruyama S. (2015). Epicardial FSTL1 Reconstitution Regenerates the Adult Mammalian Heart. Nature 525, 479–485. 10.1038/nature15372 26375005 PMC4762253

[B147] Woo Y. J. Panlilio C. M. Cheng R. K. Liao G. P. Atluri P. Hsu V. M. (2006). Therapeutic Delivery of Cyclin A2 Induces Myocardial Regeneration and Enhances Cardiac Function in Ischemic Heart Failure. Circulation 114, 206–213. 10.1161/CIRCULATIONAHA.105.000455 16820573

[B148] Xiang F.-l. Guo M. Yutzey K. E. (2016). Overexpression of Tbx20 in Adult Cardiomyocytes Promotes Proliferation and Improves Cardiac Function After Myocardial Infarction. Circulation 133, 1081–1092. 10.1161/CIRCULATIONAHA.115.019357 26841808 PMC4792775

[B149] Xin M. Kim Y. Sutherland L. B. Murakami M. Qi X. McAnally J. (2013). Hippo Pathway Effector Yap Promotes Cardiac Regeneration. Proc. Natl. Acad. Sci. U.S.A. 110, 13839–13844. 10.1073/pnas.1313192110 23918388 PMC3752208

[B150] Yang L. Xue S. Du M. Lian F. (2021). Highly Efficient MicroRNA Delivery Using Functionalized Carbon Dots for Enhanced Conversion of Fibroblasts to Cardiomyocytes. Ijn Vol. 16, 3741–3754. 10.2147/IJN.S304873 PMC8186278 34113099

[B151] Yokota T. McCourt J. Ma F. Ren S. Li S. Kim T.-H. (2020). Type V Collagen in Scar Tissue Regulates the Size of Scar after Heart Injury. Cell 182, 545–562.e23. 10.1016/j.cell.2020.06.030 32621799 PMC7415659

[B152] Zhao L. Ben-Yair R. Burns C. E. Burns C. G. (2019). Endocardial Notch Signaling Promotes Cardiomyocyte Proliferation in the Regenerating Zebrafish Heart Through Wnt Pathway Antagonism. Cell Rep. 26, 546–554.e5. 10.1016/j.celrep.2018.12.048 30650349 PMC6366857

[B153] Zhao L. Borikova A. L. Ben-Yair R. Guner-Ataman B. MacRae C. A. Lee R. T. (2014). Notch Signaling Regulates Cardiomyocyte Proliferation During Zebrafish Heart Regeneration. Proc. Natl. Acad. Sci. U.S.A. 111, 1403–1408. 10.1073/pnas.1311705111 24474765 PMC3910613

[B154] Zhao Y. Londono P. Cao Y. Sharpe E. J. Proenza C. O’Rourke R. (2015). High-Efficiency Reprogramming of Fibroblasts into Cardiomyocytes Requires Suppression of Pro-Fibrotic Signalling. Nat. Commun. 6. 10.1038/ncomms9243 PMC4579788 26354680

[B155] Zheng Q. Bao C. Guo W. Li S. Chen J. Chen B. (2016). Circular RNA Profiling Reveals an Abundant circHIPK3 that Regulates Cell Growth by Sponging Multiple miRNAs. Nat. Commun. 7, 11215. 10.1038/ncomms11215 27050392 PMC4823868

[B156] Zhou H. Dickson M. E. Kim M. S. Bassel-Duby R. Olson E. N. (2015). Akt1/Protein Kinase B Enhances Transcriptional Reprogramming of Fibroblasts to Functional Cardiomyocytes. Proc. Natl. Acad. Sci. U.S.A. 112, 11864–11869. 10.1073/pnas.1516237112 26354121 PMC4586885

[B157] Zhou H. Morales M. G. Hashimoto H. Dickson M. E. Song K. Ye W. (2017). ZNF281 Enhances Cardiac Reprogramming by Modulating Cardiac and Inflammatory Gene Expression. Genes. Dev. 31, 1770–1783. 10.1101/gad.305482.117 28982760 PMC5666675

[B158] Zhou Y. Liu Z. Welch J. D. Gao X. Wang L. Garbutt T. (2019). Single-Cell Transcriptomic Analyses of Cell Fate Transitions During Human Cardiac Reprogramming. Cell Stem Cell 25, 149–164.e9. 10.1016/j.stem.2019.05.020 31230860 PMC6684137

[B159] Zhou Y. Wang L. Vaseghi H. R. Liu Z. Lu R. Alimohamadi S. (2016). Bmi1 Is a Key Epigenetic Barrier to Direct Cardiac Reprogramming. Cell Stem Cell 18, 382–395. 10.1016/j.stem.2016.02.003 26942853 PMC4779178

[B160] Zhu D. Li Z. Huang K. Caranasos T. G. Rossi J. S. Cheng K. (2021a). Minimally Invasive Delivery of Therapeutic Agents by Hydrogel Injection into the Pericardial Cavity for Cardiac Repair. Nat. Commun. 12, 1–10. 10.1038/s41467-021-21682-7 33658506 PMC7930285

[B161] Zhu Y. Do V. D. Richards A. M. Foo R. (2021b). What We Know about Cardiomyocyte Dedifferentiation. J. Mol. Cell. Cardiol. 152, 80–91. 10.1016/j.yjmcc.2020.11.016 33275936

